# 2888. Safety and Immunogenicity of an Adjuvanted Chikungunya Virus (CHIKV) Virus-like Particle (VLP) Based Vaccine in Two Pivotal Phase 3 Trials, ≥12 Years of Age

**DOI:** 10.1093/ofid/ofad500.2471

**Published:** 2023-11-27

**Authors:** Jason S Richardson, Debbie Anderson, Jason Mendy, Sufia Muhammad, Lauren Tindale, Tobi Loreth, Sarah Royalty Tredo, Victoria Jenkins, Patrick Ajiboye, Lisa Bedell

**Affiliations:** Bavarian Nordic, Winnipeg, MB, Canada; Bavarian Nordic, Winnipeg, MB, Canada; Bavarian Nordic, Winnipeg, MB, Canada; Bavarian Nordic, Winnipeg, MB, Canada; Bavarian Nordic, Winnipeg, MB, Canada; Bavarian Nordic, Winnipeg, MB, Canada; Emergent Biosolutions, Mclean, Virginia; Bavarian Nordic, Winnipeg, MB, Canada; Bavarian Nordic, Winnipeg, MB, Canada; Emergent Biosolutions, Mclean, Virginia

## Abstract

**Background:**

CHIKV remains a significant public health concern globally. We report the results of two phase 3 trials evaluating an aluminum hydroxide adjuvanted CHIKV VLP vaccine.

**Methods:**

Two multicenter, randomized, double-blind, placebo-controlled, parallel-group trials were conducted: an adult/adolescent trial (NCT05072080) in ages 12-64 years and an older adult trial in ages ≥ 65 years (NCT05349617). Participants received CHIKV VLP vaccine or placebo as a single intramuscular dose. Immunogenicity objectives assessed anti-CHIKV NT_80_ serum neutralizing antibody (SNA) titers at selected timepoints. Seroresponse rate (SRR) was the percentage of participants who achieved NT_80_ SNA titer ≥ 100 (FDA/EMA agreed threshold). Safety outcomes were evaluated through monitoring adverse events (AEs) through Day 183.

Study overview.

**Figure:**
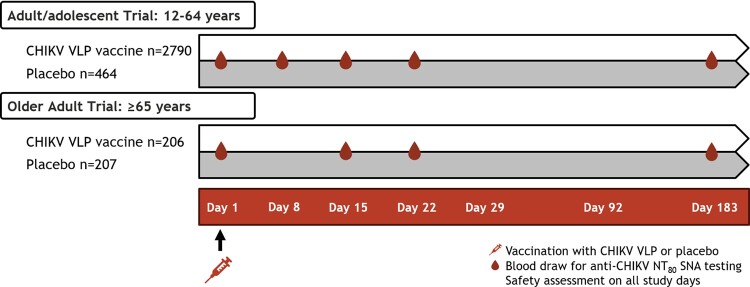

**Results:**

Adult/adolescent trial: 3254 participants (2790 CHIKV VLP vaccine, 464 placebo) were enrolled. Primary endpoints were met with a Day 22 SRR of 98% (2503/2559) for vaccine and 1% for placebo (5/424; p< 0.0001), as well as lot consistency, and superiority to placebo in geometric mean titer (GMT). A rapid antibody response was observed in the CHIKV VLP vaccine group with Day 8 SRR=47% (1169/2510) and Day 15 SRR=97% (2355/2434); responses were durable through Day 183 with SRR=86% (1967/2301).

Older adult trial: 413 participants (206 CHIKV VLP vaccine, 207 placebo) were enrolled. Primary endpoints were met with a Day 22 SRR of 87% (167/191) for vaccine and 1% for placebo (2/183; p< 0.0001), as well as by GMT. At Day 15 a rapid antibody response was observed in the CHIKV VLP vaccine group with SRR=82% (150/182).

CHIKV VLP vaccine demonstrated a favorable safety profile, and most AEs were mild to moderate in severity. The most common AEs were myalgia, fatigue, and headache.

**Conclusion:**

CHIKV VLP vaccine is the only VLP-based vaccine in clinical development for active immunization against CHIKV disease. Results demonstrate that CHIKV VLP vaccine induces a rapid and robust immune response in most people by Day 15 and through Day 183. These findings support the potential of this VLP-based vaccine to help protect individuals 12 years and older from CHIKV.

**Disclosures:**

**Jason S. Richardson, PhD**, Bavarian Nordic Canada Inc: Stocks/Bonds **Debbie Anderson, MS**, Bavarian Nordic: Employee|Emergent BioSolutions: Stocks/Bonds **Sufia Muhammad, MD**, Bavarian Nordic: Employee|Emergent BioSolutions: Stocks/Bonds **Lauren Tindale, PhD**, Bavarian Nordic: Employee **Tobi Loreth, n/a**, Emergent BioSolutions: Stocks/Bonds **Sarah Royalty Tredo, MBA**, Emergent Biosolutions: Employee|Emergent Biosolutions: Stocks/Bonds **Victoria Jenkins, PhD**, Bavarian Nordic: Employee **Patrick Ajiboye, MD**, Bavarian Nordic: Employee|Emergent BioSolutions: Stocks/Bonds **Lisa Bedell, Director Biostatistic**, Bavarian-Nordic: Advisor/Consultant|Emergent BioSolutions: Employee|Emergent BioSolutions: Stocks/Bonds

